# The centrality of social ties to climate migration and mental health

**DOI:** 10.1186/s12889-017-4508-0

**Published:** 2017-07-06

**Authors:** Jacqueline M. Torres, Joan A. Casey

**Affiliations:** 1 Robert Wood Johnson Foundation Health and Society Scholars Program, and the Center for Health & Community, University of California, San Francisco, 3333 California St., Suite 465, San Francisco, CA 94118 USA; 2 Robert Wood Johnson Foundation Health and Society Scholars Program and the Department of Environmental Science, Policy, and Management, University of California, Berkeley, Mulford Hall, 130 Hilgard Way, Berkeley, CA 94720 USA

**Keywords:** Human migration, Climate change, Mental health, Social support, Social ties, Environmental justice

## Abstract

Climate change-related hazards and disasters, known to adversely impact physical and mental health outcomes, are also expected to result in human migration above current levels. Environmentally-motivated migration and displacement may lead to the disruption of existing social ties, with potentially adverse consequences for mobile populations as well as their family members who remain in places of origin. We propose that the disruption of social ties is a key mechanism by which climate-related migration may negatively impact mental health, in particular. Existing social ties may provide social and material resources that buffer mental health stressors related to both prolonged and acute climate events. Preparation for such events may also strengthen these same ties and protect mental health. Communities may leverage social ties, first to mitigate climate change, and second, to adapt and rebuild post-disaster in communities of origin. Additionally, social ties can inform migration decisions and destinations. For example, scholars have found that the drought-motivated adaptive migration of West African Fulbe herders only occurred because of the long-term development of social networks between migrants and non-migrants through trade and seasonal grazing. On the other hand, social ties do not always benefit mental health. Some migrants, including those from poor regions or communities with no formal safety net, may face considerable burden to provide financial and emotional resources to family members who remain in countries of origin. In destination communities, migrants often face significant social marginalization. Therefore, policies and programs that aim to maintain ongoing social ties among migrants and their family and community members may be critically important in efforts to enhance population resilience and adaptation to climate change and to improve mental health outcomes. Several online platforms, like Refugee Start Force, serve to integrate refugees by connecting migrants directly to people and services in destination communities. These efforts may increasingly draw upon novel technologies to support and maintain social networks in the context of population mobility due to climatic and other factors.

## Background

Humans have long migrated due to changing climates, but have never before faced such rapid, global, anthropogenic change [1]. 2016 was the hottest year on record and recent reports find accelerating sea-level rise [2]. If high greenhouse gas emissions continue, oceans could rise by as much as 6 ft by the end of the twenty-first century [3]. Acute weather events, along with related protracted environmental and economic changes may contribute directly and indirectly to the dislocation of populations [4]. From 2008 to 2015, climate and weather-related disasters displaced an average of 22.5 million people annually [5]. However, because migration and climatic hazards are multi-causal, scholars have been hesitant to attribute any single migratory flow to climate change directly.

Nevertheless, even with successful adaptation–or activities that reduce impacts from climatic changes, such as crop substitution or new reservoir construction–climate change is expected to result in some migration above current levels [6]. Migration can also represent a positive adaptation to climate change [7] when remaining in place is no longer tenable; communities that face environmental hazards and lack the social or economic resources to move may become “trapped” in place [7].

Social ties are also a central driver of migration decisions, given that information about the costs and benefits of movement is transmitted in large part through pre-existing social networks (i.e., multiple, connected social ties) [8]. Social ties have also been linked to population health, through protective mechanisms of resource sharing (i.e., social capital) and emotional support, as well as through stress-inducing mechanisms of social and financial burden [9]. In general, populations in motion may face stressors related to the disruption of their social networks, for example, through separation from family and community members due to migration or displacement. Mobile populations may also face substantial social isolation and marginalization in destination contexts. On the other hand, they may work to maintain pre-existing social networks at a distance, by sending remittances or making phone calls to family and community members who remain in places of origin or who have also migrated [10]. These social ties may serve as a source of substantial resilience–or capacity to cope with adverse events while maintaining function, identity, and capacity to adapt, learn, and thrive–if they are characterized by high levels of trust and support (i.e., social cohesion). Social ties may have additional benefit when they provide resources for coping both emotionally and economically in the aftermath of climatic events.

Scholars and policy makers have identified numerous health consequences of climate change, including the spread of infectious disease, injury and death due to acute natural disasters, heat-related illness, as well as adverse mental health outcomes [11]. Discussion of the health consequences of climate *migration* has been more limited, but has included altered distribution of infectious diseases [12], increased violence [13], and reduced access to healthcare [5].

**Fig. 1:**
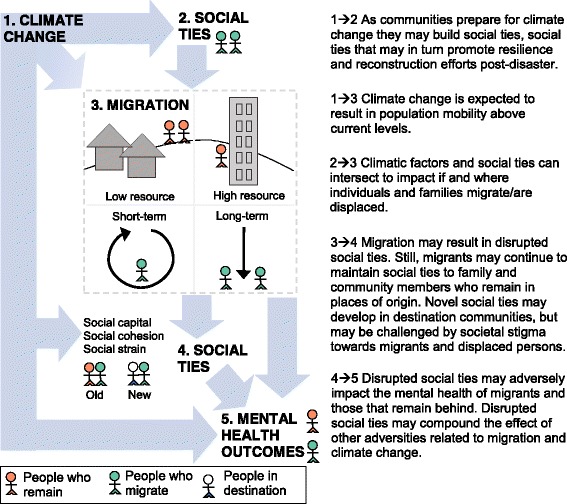
Conceptual Framework for the Role of Social Ties in Climate-Related Migration and Mental Health

Nevertheless, public health requires a richer understanding of the role of the social environment on health before, during, and after climate migration. In this commentary, we propose a framework that considers social ties as both drivers and modifiers of climate migration *and* the health outcomes of migrants (Fig. 1). We focus our commentary on mental health, given the strong empirical support for the link between social ties and mental health outcomes and the relative absence of literature discussing mental health in the context of climate change [14]. Mental health, defined by the World Health Organization as an overall state of wellbeing that includes the ability to cope with daily stressors and make contributions to one’s family and community, is a critical component of population wellbeing and productivity [15].

Throughout the commentary we provide examples of research on each component of our conceptual framework (Fig. 1). We draw on a broad body of evidence, including examples that are not directly related to climate change, but that may provide an indication of what might occur in the aftermath of acute and protracted climatic events. This includes evidence of health and mobility outcomes after earthquakes, or in the context of labor or conflict-related migration flows. While we incorporate examples from across the globe, we note that vulnerability to climate change and climate migration is heterogeneous both across and within country contexts. Low and middle-income countries are disproportionately impacted by climate change, although they have received relatively little economic benefit from the activities that have fueled global warming [16]. Low-income, rural populations are also at greater risk for the loss of livelihoods due to climate change, while their low-income urban counterparts face risk due to the fragility of housing and infrastructure in poor urban neighborhoods across the globe. We elaborate below on specific population groups that may be more vulnerable to adverse mental health outcomes as the result of climate migration and disrupted social networks. Finally, we discuss the practical implications of the research on climate change, social ties, migration, and mental health. In particular, we highlight the potential for leveraging novel technology in order to help maintain social ties in the context of climate migration.

## The link between climate change and social ties

Climate change is expected to impact social relationships between family and community members (Fig. 1, path 1 → 2). In large part, these effects are expected to be negative. Reduced natural resources may lead to increased competition among community members for food, water, and livelihoods [17]. Acute climatic events may result in the loss of family dwellings and community meeting places, leading to reduced opportunity for social interaction, and increased social strain [14]. On the other hand, there is a strong call to acknowledge the role of individual and community resilience and adaptation in the face of climate change [18]. In particular, climatic events might have the capacity to strengthen family and community ties, provided there are opportunities to rebuild. Adaptation efforts prior to the onset of acute disasters or the most damaging effects of protracted climate changes may also provide opportunities for strengthening social ties. For example, Mathbor [19] describes that the Cyclone Preparedness Program in Bangladesh provides ongoing opportunities for interaction and trust-building among local volunteers, while also contributing to community preparedness for frequent cyclones. Evidence from other settings suggests that supportive community social ties (i.e., community social cohesion) serve as a source of resilience in the context of protracted and acute disasters. Social ties can facilitate the exchange of resources (e.g., food, shelter) and information (e.g., early warnings regarding environmental risks), and increase the likelihood of disaster preparedness [20, 21].

## The importance of climate for migration

Although migration patterns are complex and multi-causal, environmental conditions have always been an important driver of individual and population-level mobility [22] (Fig. 1, path 1 → 3). Nevertheless, rapid anthropogenic change in climate patterns and an increase in climatic events are likely to contribute to population mobility above current levels. In a recent example, Syria experienced the most severe drought in recorded history from 2006 through 2007, the result of both cyclical and long-term declines in precipitation and groundwater. The drought contributed to the collapse of small and mid-scale agriculture and the migration of 1.5 million Syrians from rural areas to urban peripheries [23]. While no certainty exists regarding the direct, causal effects of climate change on migration, in this and other examples non-cyclical climatic change is a contributing factor, and climate-related resettlement has commenced globally [24].

Climate-related population mobility may take many forms. Circular migration, defined as fluid movement between countries, may entail short- or long-term moves and is often linked to labor demands in places of destination [25]. Temporary or permanent movement of individuals or entire families may occur as the result of either protracted or acute climatic events. In the most extreme form, planned permanent resettlement may lead to the relocation of entire communities, as with low-lying coastal or island populations [26]. These migration patterns are not mutually exclusive: in the aftermath of the same climatic event, some members of a community may temporarily move while others may permanently resettle elsewhere if the economic and social benefits of staying in their new location outweigh the perceived benefits of returning home [27]. Population mobility may also occur within countries or internationally, although climate-related migration has been linked primarily to internal (i.e., within-country) migration [28].

Finally, environmental factors may intersect with other micro- (e.g., family) and macro-level (e.g., national policy) circumstances to influence population mobility [29]. We discuss the intersection between environmental and social factors below. However, we note that political, economic, and demographic factors may also critically influence population vulnerability to climate change, and environmental migration patterns. In charting the aftermath of the recent historic drought in Syria, Kelley and authors [23] describe that the collapse of much of the country’s agricultural production. Mass rural-to-urban internal migration also occurred in the context of the Syrian government’s restrictive economic policies that exacerbated food shortages, as well an influx of Iraqi refugees that may have strained resources. The intersection of climate change with these factors contributed to the Syrian civil conflict, which has in turn triggered mass population displacement. In another example from Pakistan, two consecutive years of flooding (2010–2011) combined with deforestation and a nascent national disaster response system, resulted in mass displacement of 150,000 people to relief camps [30].

## The importance of social ties for climate-related migration

Scholars have long emphasized the influence of social ties on population mobility patterns (Fig. 1, path 2 → 3). Individuals with prior migration experience serve as a source of information about the migration passage and expected outcomes (e.g., the availability of jobs) [8]. The desire to reunify with family or community members may also motivate migration. However, there is increasing recognition of the intersection of social and environmental migration drivers (Fig. 1, path 1,2 → 3). For example, in West Africa, Bassett and Turner found that drought-motivated adaptive migration of Fulbe herders from the Sudano-Sahelian to the Sudano-Guinean region only occurred because of the long-term development of social networks between migrants and non-migrants through trade and seasonal grazing [31]. In another example, a study of the effect of rainfall patterns on Mexican migration to the U.S. found that Mexican residents were more likely to migrate after years of drought *only* if they lived in regions with historically high levels of U.S. migration [32]. Individuals from these regions likely had strong ties to migrant family or community members, which facilitated the process of migration and lowered the economic barriers to doing so. For individuals living in regions with lower levels of prior U.S. migration–and likely fewer social ties to individuals with migration experience–drought actually predicted *lower* out-migration, given that drought likely reduced the economic resources necessary for migration.

Social ties can influence population mobility beyond migration for labor or family reunification. In an example of environmentally-motivated migration, by the early 1930s, over 60,000 people from Oklahoma had resettled in California as part of the Dust Bowl migration [33]. These early migrants guided new arrivals to find housing, employment, and to integrate with the host population. Similarly, during acute weather events, individuals may decide whether and where to move based on information gathered from people in their social networks [34].

The relationship between social ties and environmental migration is not always direct and positive. It may be complicated by specific characteristics of social relationships and the ability of these ties to provide assistance. Findings from research predicting environmentally motivated internal migration within Bangladesh suggest that having family and community members with previous migration experience was associated with increased likelihood of making temporary rather than permanent moves [35]. However, when respondents reported receiving financial assistance from family members living in destination communities they were more likely to settle permanently rather than engage in temporary, circular migration patterns.

## Migration and disrupted social ties

The very same social ties that shape population mobility may be disrupted in the process of migration or displacement (Fig. 1, path 3 → 4). Family and community members may face temporary or long-term separation if some move while others remain in places of origin [36]. In other cases, the process of fleeing acute disasters and conflict may disperse family and community members. Once separated, refugees and other displaced persons may face substantial administrative barriers to reunifying even with immediate family members in other countries. For example, documents that serve as evidence of biological family connection and dependency (e.g., birth certificates) may be lost or destroyed in acute disaster settings [37]. Even if immediate family members can migrate together or to reunite after separation, extended or non-biological family and community members may be geographically dispersed as the result of administrative assignment to different destination countries and communities, as with refugee resettlement “lottery” programs.

Migrants may maintain a connection to extended family and community members by communicating through letters, telephone and, increasingly, mobile applications that allow for affordable video-conferencing and text messaging [10]. These ongoing social connections may be paired with continued economic connection; migrants remitted $601 billion dollars in 2015 [38]. While these remittances play a critical role in providing for the basic needs of those who remain in places of origin, they also can have important symbolic and emotional value for connecting those who send and receive them [39]. Finally, migrants and displaced persons may in some cases make return visits to places of origin, although the option to return may be limited by ongoing conflict in places of origin or political barriers to mobility (e.g., for undocumented migrants). On the other hand, places of origin may no longer be safe to return to, or may no longer exist, as with low-lying island and delta regions that are expected to be submerged by rising sea levels over the course of the twenty-first century [26, 40].

In addition to facing the potential disruption of extended family and community networks, migrants and displaced persons face challenges to developing novel social ties in destination communities. Migrants and displaced persons often face societal stigma and marginalization as well as social isolation. Social marginalization may compound the lack of legal protections currently afforded to those displaced by climatic factors. Indeed, those displaced by climate change are not formally protected under international refugee law [41] and may face limited options for political and social integration into destination societies.

## Migration, disrupted social ties, and adverse mental health

Regardless of the stimulus for migration, recent literature finds some evidence of poorer mental health and wellbeing among migrants and displaced populations relative to populations of non-movers in countries and communities of origin [42–44]. For example, a cross-national study found that Mexican migrants to the U.S. had higher levels of depression compared to their counterparts who never left Mexico [45]. Migration may contribute to adverse mental health outcomes through a number of potential mechanisms, including exposing populations in motion to traumatic circumstances along the migration passage and discriminatory attitudes and treatment in places of destination. Scholars have also noted that there may be unique, adverse mental health consequences of losing access to the natural landscape [46]. Albrecht and authors have captured this phenomenon with the term “solastalgia” or a sense of distress and melancholy related to the environmental destruction of one’s home [47].

Migration may also adversely impact mental health by disrupting social and community connections (Fig. 1, path 4 → 5). Separation from one’s close family members due to a disaster or conflict may be an acute stressor with both short and long-term impacts on mental health outcomes. Disrupted social ties may contribute to adverse mental health outcomes through mechanisms of social isolation and reduced social and material (e.g., informational, financial) support [48]. Separation from members of one’s origin family or community may also lead to a reduced sense of belonging within a familial, regional, or ethnic network, which may contribute to poorer mental health [49]. Migration-related separation may compound the impact of adverse experiences related to climate change, including the trauma of acute weather events, food and water insecurity, the loss of one’s land or home, and reduced social cohesion as the result of increased competition for basic resources such as food and water [14, 50].

Nevertheless, the impact of migration on mental health may vary. A study of Tongan migrants to New Zealand, with rigorous attention to migration selection based on mental health (i.e., the possibility that individuals who move have systematically different pre-migration mental health than non-movers), found evidence of significantly *improved* mental health among migrants relative to those who remained in Tonga [51]. Although, in this case, all migrants moved with their immediate family members and many explicitly cited an improved “social life” as their motivation for migration. The mental health impacts of population mobility may, therefore, depend on how disruptive moves are to existing social ties, in addition to other factors such as whether moves are temporary or permanent, and the potentially traumatic exposures endured prior to or during migration or displacement.

Social cohesion represents a key component in supporting psychological outcomes of populations affected by disasters and displacement. Using data collected both before and after the 2011 Tohoku earthquake, Hikichi and colleagues found that higher levels of pre-earthquake social cohesion were protective of post-disaster adverse mental health outcomes [52]. Likewise, individuals who were both displaced from their homes after Hurricane Katrina and lived in counties with low social cohesion, measured as an aggregate of individual perceptions of community trust and support, had significantly higher odds of past-month depression compared to those who experienced only displacement or low social cohesion [53]. Even in the absence of climate-related migration or resettlement, these examples underscore the benefit of strengthening social ties. 

## Migration, disrupted social ties, and the mental health of those who remain

There may be adverse mental health consequences of population mobility for those who do not move (Figure 1, path 4 → 5). In some cases, entire households may migrate or be displaced. In other cases, some members migrate temporarily or permanently to seek out alternative livelihoods while some family members remain behind. Evidence is mixed on the effect of family member migration or displacement on the health of those who remain. On the one hand, the out-migration of family members has been linked to increased depression and other mental health disorders for those who remain behind. For example, the Three Gorges Dam in China, built to provide renewable energy, forced over a million people to relocate and degraded the surrounding environment. Researchers found evidence of a significant increase in depression in those who remained behind during the Three Gorges Dam project [54]. The results of the same analysis suggested that the effects of relocation on depression for those who remained may have been partially mediated by changes in personal resources, including household income and perceived social support. On the other hand, researchers have also reported no change in depression or even *lower* levels of depression as the result of family member out-migration in other contexts [55].

The adverse mental health consequences of migration-related separation for those who remain in places of origin may be compounded by other climate-related vulnerabilities. These may include the loss of livelihoods, lack of access to food and clean water, and civil conflict. Environmental changes may degrade familiar landscapes, cultural heritage sites, and alter patterns of socialization, with negative implications for mental wellbeing even among those who do not migrate [47, 56].

In the context of climate change, migration-related economic opportunities may become of increasing importance to those who remain in places of origin as livelihoods, food, and water supplies are threatened. Remittances sent back to communities of origin may support climate adaptation [57]. For example, migrants and “hometown” organizations can support water and irrigation projects in places of origin that may help individuals cope with the effects of climate change. Some of these development projects, such as renewable energy efforts, may have co-benefits for climate change mitigation.

## Implications for practice and research

There are important practical implications that emerge from an understanding of the centrality of social ties for population mobility and population mental health. International aid organizations are already involved in re-connecting family members who have been separated by large-scale population mobility or natural disasters. Historically, the Restoring Family Links program through the International Committee of the Red Cross has transmitted letters between refugees, displaced persons, and their family members [58].

Mobile technologies have become increasingly critical for refugees and migrants seeking to reunite with family members in these acute disaster contexts [58, 59]. For example, Google People Finder was used to locate missing individuals in the aftermath of the 2011 earthquake in Japan. The United Nations Children’s Fund (UNICEF) has developed RapidFTR (or Rapid Family Tracing and Reunification), an open-source mobile phone application to help reunite children with their caregivers in disaster settings (https://github.com/rapidftr/RapidFTR/wiki). Another online platform, the Refugee United Project, allows individuals to register in and search through a database to trace missing family members. Other technological advances, like unmanned aerial vehicles that provide post-disaster communications networks [60], may also support maintenance of social ties.

Collaboration between international organizations, national governments, and the private sector will further serve to incorporate rapidly developing technological advances into efforts to re-connect mobile populations [58] as well as providing long-term resources for maintaining everyday social and economic ties, given the potentially protracted nature of many migratory flows. For example, the World Bank has partnered with national authorities to understand the potential for leveraging mobile technologies to decrease the excess cost of sending remittances, e.g., via smartphone applications that allow migrants to compare international money transfer fees across multiple financial agencies [61]. Again, remittances represent an important economic connection between migrants and their family and community members who remain in places of origin, but also have important symbolic value for family members seeking to maintain social connection in the aftermath of migration [39]. These collaborative efforts should also include evaluation, as there is little empirical evidence of the effectiveness of technology-driven strategies for maintaining and/or strengthening social ties disrupted due to migration or displacement.

Climate change may result in other large infrastructure adaptation projects like sea walls and dams that displace entire communities. Investments in enhancing community social cohesion would benefit population mental health both before and after individuals move. Evidence from Three Gorges Dam suggests that even *anticipating* the large-scale displacement of one’s community is associated with significantly increased depression [62]. While other cases of planned resettlement have thus far been rare, the U.S. Department of Housing and Urban Development has awarded its first grant aimed at resettling an island-dwelling community in Louisiana. One of the guiding principles for carrying out this voluntary resettlement is that “activities must include building and bridging social networks as part of the process and outcome,” which if followed should benefit the mental health of relocated individuals [63]. This case provides a critical opportunity to track the impact of planned climate-related resettlement on social ties and mental health, and to evaluate policies aimed at maintaining social networks throughout the resettlement process.

Even among those who have been temporarily displaced by acute climate events, efforts may aim to maintain and strengthen community social ties. For example, mental health interventions targeting those who survived the 2004 tsunami in India included individual therapy, but also engaged community members in re-building and activities that provided survivors with a sense of purpose (e.g., caring for children) and generated social cohesion [64]. Ongoing efforts must implement and evaluate programs that strengthen social cohesion in the face of lost livelihoods and population mobility.

For those who are permanently displaced, public and private-sector efforts may support social integration within destination communities. Some qualitative evidence suggests that mobile technologies may be critical for facilitating social connection between newly arrived refugees and those in the host societies [65]. Several online platforms serve to integrate refugees into destination communities. With the slogan “connecting people, creating networks,” Refugee Start Force (https://refugeestartforce.eu/) uses their online platform and groups on networking sites like Facebook and LinkedIn to connect refugees with professional opportunities in the Netherlands. Airbnb, the popular online lodging rental site, has created a response tool that when activated in the aftermath of disasters allows hosts to open their homes to displaced persons free of charge ​(https://www.airbnb.com/welcome). In Germany, Help To (http://helpto.de/en) offers a range of services to refugees including offers of and requests for legal advice, donations, and transportation. Nevertheless, to our knowledge, there is currently no empirical evidence of the effectiveness of these programs. Moreover, these novel efforts should not supplant public policy efforts to integrate migrants and refugees, as there is evidence of the causal effect of naturalization on increased social integration, particularly for the most marginalized migrants [66].

Strengthened social ties between migrants and those living in destination or settlement communities might have additional important co-benefits for population health. For example, social isolation is a key risk factor for heat-related health problems. The Centers for Disease Control and Prevention guidelines for preventing heat-related illness include buddy systems and formal check-ins with vulnerable persons; efforts that draw in part upon existing social ties among family and neighbors [67]. Such network-building to address climate change might also create greater community social cohesion, and consequently, improve population mental health.

## Special considerations for vulnerable populations

Certain populations are expected to face a disproportionate share of adverse health outcomes resulting from climate change and increased population mobility. Climate change itself may also compound population vulnerability over time as feedback loops erode economic, social, and political conditions [68]. Indigenous communities face particular threats related to food insecurity and loss of native lands, although local knowledge may aid adaptation [69, 70]. Older adults in general face greater health risks in the context of climate change and older migrants may also experience high levels of social isolation in reception communities and reduced social support in places of origin if family members migrate [71]. Children also face considerable vulnerability in the context of climate migration, including potential separation from primary caregivers and the disruption of educational trajectories, which may, in turn, have lasting, adverse mental health consequences [72].

For those living in poverty, efforts to facilitate continued social and economic ties among mobile populations are critical. Remittances sent from migrants to origin communities provide resources to those not covered by formal, social safety net programs [73]. On the other hand, the obligation to remit may represent a substantial financial burden for migrants, many of who are laboring in low-wage sectors. While there is some evidence linking remittance sending to improved mental health among remittance-senders [74], others have reported the opposite effect [75]. Adverse mental health effects of remittance sending appear particularly strong among women, who may bear a larger burden of maintaining family networks and providing emotional and financial resources to family members who have remained behind [49].

## Conclusion

Although estimates remain uncertain, climate change is expected to result in human migration above current levels. This increased population mobility is a potentially important mechanism linking climate change and population mental health. One pathway by which environmentally-motivated migration may negatively impact mental health is through the disruption of existing family and community ties. The disruption of these social ties may adversely impact migrants or displaced persons, potentially compounding the effects of other adversities, including traumatic experiences before and during the migration passage. Disrupted social ties resulting from out-migration may also impact those who remain in communities of origin, including populations already facing the loss of livelihood, natural landscapes, and cultural heritage sites as the result of climate change. Reconnecting and strengthening social ties in both origin and destination communities may increase resilience and buffer some of the adverse mental health impacts of acute and protracted climate events. Relevant strategies may increasingly involve leveraging novel technologies. Nevertheless, there is a need for rigorous evaluation of the effectiveness of these technologies for improving social cohesion and/or mental health for mobile populations and their family members. In addition, strategies to promote social connectedness should factor in the potential for social and economic strain among migrants and displaced persons. In particular, the obligation to remit to family and friends who remain may represent a substantial financial burden for migrants.

The costs and effort related to supporting migrants and displaced persons should reflect global responsibility for the consequences of climate change. Indeed, environmental injustice led to anthropogenic climate change. Greenhouse gas emissions that drive climate change have simultaneously propelled the economic growth of wealthy countries and individuals [57]. Now, developing countries and the poor will experience the greatest impacts, including increased risk for displacement, disrupted social ties, and adverse mental health consequences. It is a matter of social justice to invest in the mental health of these populations in motion. This includes implementing strategies that support and strengthen existing social ties as well as efforts that enhance social integration within destination communities for migrants who may be permanently displaced from places of origin.
